# Perlecan/HSPG2 and matrilysin/MMP-7 as indices of tissue invasion: tissue localization and circulating perlecan fragments in a cohort of 288 radical prostatectomy patients

**DOI:** 10.18632/oncotarget.7197

**Published:** 2016-02-04

**Authors:** Brian Grindel, Quanlin Li, Rebecca Arnold, John Petros, Majd Zayzafoon, Mark Muldoon, James Stave, Leland W. K. Chung, Mary C. Farach-Carson

**Affiliations:** ^1^ Department of BioSciences, Rice University, Houston, TX 77005, USA; ^2^ Biostatistics and Bioinformatics Research Center, Samuel Oschin Comprehensive Cancer Institute, Cedars Sinai Medical Center, Los Angeles, CA 90048, USA; ^3^ Emory University Departments of Urology, Pathology and Laboratory Medicine and Hematology and Medical Oncology, Atlanta, GA 30322, USA; ^4^ The Atlanta Veteran Affairs Medical Center, Decatur, GA 30033, USA; ^5^ Department of Pathology, University of Alabama at Birmingham, Birmingham, AL 35233, USA; ^6^ Strategic Diagnostics Inc., Newark, DE 19702, USA; ^7^ Uro-Oncology Research Program, Samuel Oschin Comprehensive Cancer Institute at Cedars-Sinai Medical Center, Los Angeles, CA 90048, USA; ^8^ Department of Bioengineering, Rice University, Houston, TX 77005, USA; ^9^ Romer Labs Technology, Inc., Newark, DE 19713, USA; ^10^ CD Diagnostics, Claymont, DE 19703, USA

**Keywords:** perlecan, matrilysin, prostate cancer, metastasis, invasion

## Abstract

Prostate cancer (PCa) cells use matrix metalloproteinases (MMPs) to degrade tissue during invasion. Perlecan/HSPG2 is degraded at basement membranes, in reactive stroma and in bone marrow during metastasis. We previously showed MMP-7 efficiently degrades perlecan. We now analyzed PCa tissue and serum from 288 prostatectomy patients of various Gleason grades to decipher the relationship between perlecan and MMP-7 in invasive PCa. In 157 prostatectomy specimens examined by tissue microarray, perlecan levels were 18% higher than their normal counterparts. In Gleason grade 4 tissues, MMP-7 and perlecan immunostaining levels were highly correlated with each other (average correlation coefficient of 0.52) in PCa tissue, regardless of grade. Serial sections showed intense, but non-overlapping, immunostaining for MMP-7 and perlecan at adjacent borders, reflecting the protease-substrate relationship. Using a capture assay, analysis of 288 PCa sera collected at prostatectomy showed elevated levels of perlecan fragments, with most derived from domain IV. Perlecan fragments in PCa sera were associated with overall MMP-7 staining levels in PCa tissues. Domain IV perlecan fragments were present in stage IV, but absent in normal, sera, suggesting perlecan degradation during metastasis. Together, perlecan fragments in sera and MMP-7 in tissues of PCa patients are measures of invasive PCa.

## INTRODUCTION

Prostate cancer (PCa) in the United States alone presents an estimated 233,000 new cases and over 29,000 deaths a year. While organ confined PCa has a five-year survival rate of nearly 100% in the US, metastasis reduces this Figure to near 28% [1]. Further understanding of the mechanisms of PCa metastasis is vital to identify the subset of PCa patients who will progress rapidly or relapse after hormone deprivation therapy. The development of indices of likely invasion using sera and tissues from PCa patients could assist with the differentiation of indolent from aggressive forms of cancer for improved treatment upon disease progression.

Locally invasive PCa cells depend on proteases, such as matrix metalloproteinases (MMPs), to move through the extracellular matrix (ECM) to reach the final site of metastasis [2]. PCa has a predilection for metastasis to bone marrow, where osteoblastic/sclerotic lesions are hallmarks [3]. To arrive at the sites of distant metastasis, PCa cells must degrade and cross five separate tissue borders containing the large heparan sulfate proteoglycan (HSPG) perlecan/HSPG2 [4, 5], a multimodular five domain ECM proteoglycan with N-terminal heparan sulfate (HS) and/or chondroitin sulfate (CS) glycosaminoglycan (GAG) chains [6]. By interacting with a wide variety of growth factors and ECM components, perlecan modulates many fundamental biological processes including angiogenesis, differentiation, growth, and early development [7]. Given perlecan's actions, it is not surprising that it is involved in the pathogenesis of cancer [8]. Perlecan is a major constituent of the glandular basement membrane (BM), the reactive stroma that surrounds the tumor in response to cancer-produced and growth factor-elicited signals, the vascular BM at sites of both intravasation and extravasation, and as a reticular network in the bone marrow metastatic niche [4, 9, 10]. With respect to PCa, we showed previously that perlecan expression is upregulated as a part of the tumor necrosis factor α-induced desmoplastic response to expanding and invading PCa tumors [5]. Deposition of perlecan in response to cancer invasion may be universal, because it also was found in the invasion fronts of oral squamous cell carcinoma cells [11].

This study first investigated the tissue and serum levels of perlecan and matrilysin/MMP-7. MMP-7 previously was found to cleave perlecan regardless of how it is presented: within intact BM, fully decorated with HS/CS chains, or as a recombinantly produced subdomain of domain IV. Notably, degradation of perlecan by MMP-7 completely reversed its ability to trigger PCa cell clustering, leading to tumor dyscohesion and favoring a migratory single cell phenotype [12]. MMP-7, a small zinc dependent endopeptidase with a wide array of substrates [13], is the best catabolizer of perlecan among several proteases that have been tested [12, 14, 15]. In aggressive PCa cancers, MMP-7 is upregulated in relation to its natural inhibitor, tissue inhibitor of MMP 1 (TIMP-1) [16, 17], and PCa overexpressing MMP-7 displays increased invasiveness in a murine model of metastasis [18]. Likewise, perlecan levels are increased in PCa tumor specimens over normal prostate [19]. We thus investigated the presence and levels of perlecan and MMP-7 in a cohort of 288 PCa patients undergoing radical prostatectomy from which both tissue and serum were collected using a standardized procedure. The working hypothesis of this investigation was that perlecan and MMP-7 levels and localization are coordinately regulated in the tumor microenvironment, and a pathophysiologic relationship between these factors may predict the risk of invasion and metastases in a subset of PCa patients.

β2-microglobulin (β2M) is best known as a component of the major histocompatibility class 1 antigenic presentation complex [20], but also is a factor implicated in inducing epithelial mesenchymal transition (EMT) and osteomimicry in PCa [21]. It also plays a role in tissue inflammation, homeostasis and ECM turnover in reactive stroma by increasing production of degradative enzymes, including MMPs [22]. Overexpression of β2M in PCa cells enhances bone metastasis in a mice model [23]. Because it can be a modulator of MMP activity, we also examined the levels of β2M in serum and tissue of PCa patients at the time of prostatectomy to determine if a triad relationship might exist with perlecan and MMP-7 in well-characterized tissue specimens and sera that can offer prognostic indicators of PCa grade, invasion potential and likelihood of distant metastasis.

## RESULTS

### Study design

**Figure 1:**
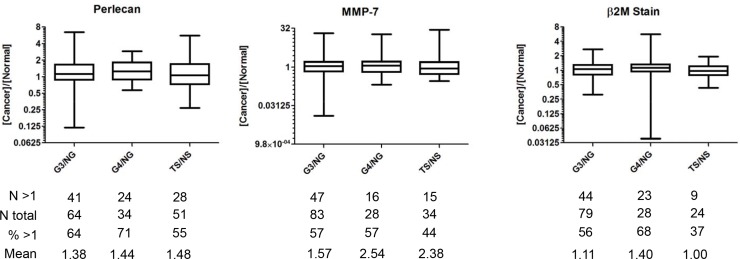
Ratios of stain concentration for perlecan, MMP-7, and β2-microglobulin in TMA sections Gleason grade 3 and 4 over normal gland (G3/NG and G4/NG), and tumor stroma over normal stroma (TS/NS) ratios were assessed in TMA sections from the same patient with N showing the number of cases examined for each (N Total). The number of cases with ratios above 1 (greater stain concentration in the cancer core) (*N* > 1), percent of cases above 1 (% > 1), and the mean ratio value for each type are shown below each graph.

**Figure 2:**
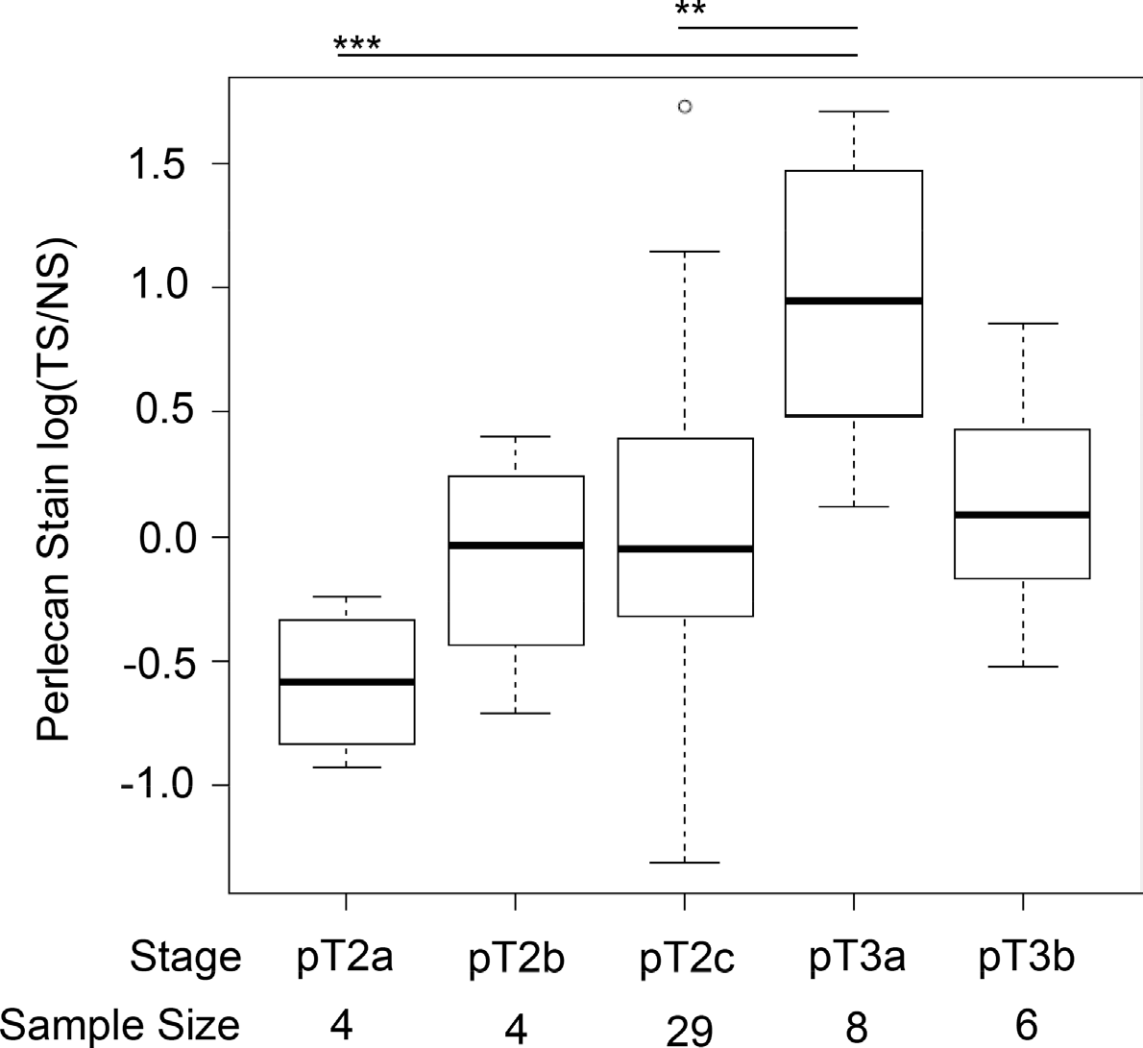
Perlecan stain within patients is increased in stage pT3a Box plots of perlecan ratiometric stain for tumor stroma over normal stroma, TS/NS, separated into different clinical stages with the respective sample size. The ratio is significantly higher for pT3a in comparison to pT2c and PT2a. A ** indicates a *p* value < 0.005 and *** is a *p* value of approximately 0.

The study design we used is shown in Supplementary Figure S1 and described further in Materials and Methods. A cohort of 300 patient subjects undergoing a prostatectomy had serum collected. A subset of these patients (157) had cores removed each from the cancerous region and the normal region of the paraffin embedded resected prostate to create a tissue microarray (TMA) in serial section. TMAs were stained for perlecan, MMP-7 and β2M in series, after which the arrays were imaged, allowing a pathologist to circle regions of interest (ROI) for various tissue/annotation types. The ROIs were quantified for stain amount that ultimately became a stain “concentration”. Concentration values were analyzed with respect to grade, stage, and matched serum from the 157 patients from whom both tissue and serum were provided. Additionally, a new ratiometric value [cancer]/[normal] was derived from two tissue cores, a cancerous region and normal region, each taken from the same resected prostate, but not immediately adjacent (Figures 1 and 2).

### Perlecan, MMP-7 and β2M stain with formalin fixed paraffin embedded tissue

Staining for perlecan in paraffin fixed tissue previously has been difficult, leaving many studies to resort to frozen tissue where tissue architecture is often difficult to preserve. Hence, initial effort in this study was devoted to developing a reliable stain for perlecan on paraffin fixed tissue such that it could be used with both archived and new tissue blocks. The perlecan antibody used in this TMA was verified by the Human Protein Atlas project to work on formalin fixed paraffin embedded (FFPE) tissue (http://www.proteinatlas.org/ENSG00000142798/antibody), however, the antigen retrieval protocol was modified to allow visualization of stromally deposited perlecan by pre-digestion with hyaluronidase. The stain we obtained generally was lighter than that from acidic heat-induced epitope retrieval, however, there was less extracellular loss of perlecan resulting in a more revealing stain overall.

**Figure 3:**
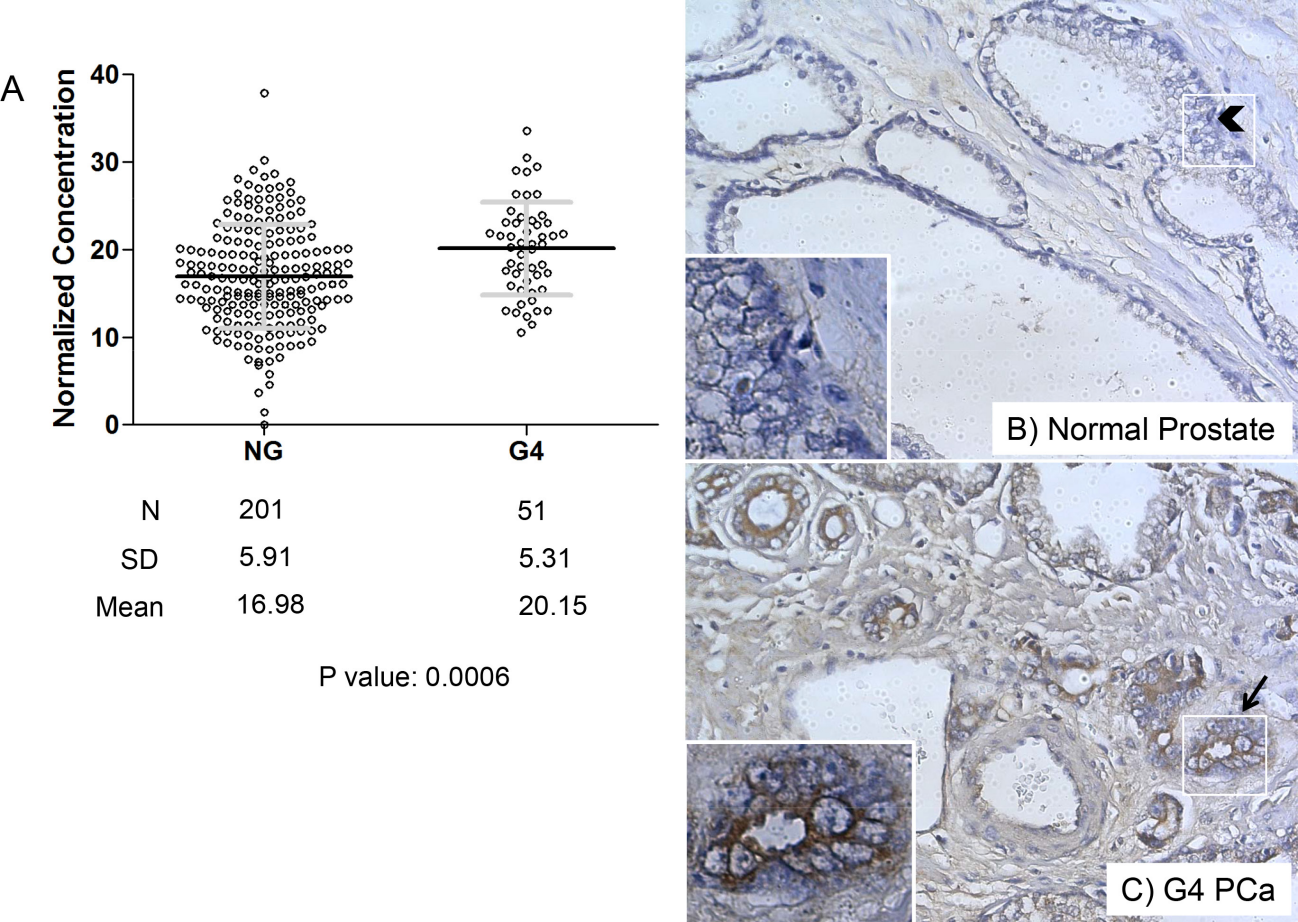
Perlecan concentration is increased in Gleason grade 4 (G4) tissue over normal gland (NG) tissue in the TMA (**A**) Graph comparing G4 tissue concentration to NG concentration with respective annotation case numbers (N), mean and standard deviation (SD). An unpaired parametric two tailed *t*-test was performed to test the difference between the means (see text). The *p*-value was 0.0006 (***). (**B**) Representative image of normal gland core with low perlecan stain with an arrowhead indicating absence of perlecan stain between cells. (**C**) Representative image of G4 core with perlecan stain with an arrow highlighting pericellular staining in a cancerous gland. Both B and C are 10X objective.

Supplementary Figure S2 displays the staining patterns we obtained with the anti-perlecan antibody and various controls to verify the specificity of the primary and secondary antibody detection systems, and also to show a lack of background interference with analysis. Figure S2A shows a higher cell-associated perlecan stain, with only some staining in the surrounding matrix. Cell-associated staining was less commonly seen than the stromal pattern in Figure S2B, where perlecan stain was associated with territorial matrix while largely absent in the gland. Extensive digestion with proteinase K led to epitope destruction, and demonstrated the low background associated with the perlecan primary antibody (Figure S2C), and the anti-green fluorescent protein (GFP) antibody shows the low background associated with the secondary visualizing system (Figure S2D). The procedure typically stained the perlecan-dense vasculature heavily, as expected, but in general the thin glandular BM was difficult to visualize in many sections, attributable either to under retrieval or epitope blockade. The stain amount of stromally deposited perlecan varied in intensity and was different in various sections, which was quantitated for the total specimen pool in Figure 3.

The antibody used for detection of MMP-7 was specific for both the latent and active forms of the enzyme, meaning the protease is not necessarily active when stain is present. The antibodies for MMP-7 and β2M are commonly used in FFPE tissue immunohistochemistry (IHC), and yielded staining patterns similar to that found in literature [16, 24].

### Expression of perlecan, MMP-7, and β2M

Protein expression patterns of perlecan, MMP-7, and β2M next were assessed by TMA in serial sections of the entire specimen pool (See Table 1, Supplementary Figure S1 and text in Materials and Methods). Each prostate of the 157 used in this study supplied two tissue cores; a core with cancerous region and a core with a normal region as assessed histologically. We produced a new value comparing the cancerous region with the normal region stain concentrations ‘[cancer]/[normal]’ for Gleason grade 3 and 4 (G3/4) over normal gland (NG). We also created a value for tumor stroma (TS) over normal stroma (NS) by examining only the stroma in the cancerous core compared to that in the normal core. This created three new ratiometric values: G3/NG, G4/NG, and TS/NS. If a value was reported to be greater than 1.0, the tissue had more stain concentration in cancerous tissue than normal tissue, and the opposite was the case if the value was lower than 1.0. Figure 1 shows a standard box plot for the expression profiles of perlecan, MMP-7, and β2M based upon analysis of tissues from the subjects in this study. We did not include G5/NG values because the low sample size (*N* = 3, *N* = 4) was insufficient. Below the graph is the number (*N* total) of mined annotations, the number of values above 1.0 (*N* > 1), the percentage of values above 1.0 (% > 1), and the mean value for each ratiometric comparison group. This value normalizes any slide-to-slide stain variability and accounts for inherent subject variability. If no variation in marker expression existed between normal and cancerous tissue, the mean would be 1.0 and the % > 1 would be 50%. As shown, perlecan mean [cancer]/[normal] value for G3, G4 and TS show an approximately 40–45% increase in cancer tissue stain over normal. Additionally, a majority of the mean values are above 1.0 (55–71%). MMP-7 showed the same trend as did perlecan, but the specimen to specimen variation was higher than that seen for perlecan (∼8X). MMP-7 values varied from near 32 to below 0.031 (MMP-7 G3/NG), meaning some glands had as much as 32 times more or alternatively 32 times less stain in G3 versus its normal tissue counterpart. MMP-7 expression also demonstrated the highest [cancer]/[normal] mean value deviation from 1.0 with 2.54 and 2.38 for G4/NG and TS/NS, respectively. The mean value is shifted up greatly for MMP-7 in TS/NS by a few “superexpressers”, because only 44% of total values are above 1.0, yet the mean is high at 2.38. The mean value shifts for β2M are modest in comparison to the other biomarker stains, save for G4/NG (1.40). In fact, the mean for TS/NS was almost exactly 1.0, indicating lack of a stromal response altering β2M expression. Overall, these findings demonstrate the expression variability and heterogeneity in staining patterns between cancer and normal tissue within the patient specimen set that we studied. In general, the staining concentration for perlecan and MMP7 for the entire tissue pool that we examined was higher in PCa versus normal for tissues of both glandular and stromal origin.

**Table 1:** Patient pool at prostatectomy

CHARACTERISTICS	ELISA	TMA
Total Subjects	288	157
Gleason sum 6	113	38
Gleason sum 7	160	109
Gleason sum 8	6	3
Gleason sum 9	9	7
Biochemically Recurrent	30	26
Average Time to Recurrence (months)	12.7	12.9
Stage 1	3	2
Stage 2	232	115
Stage 3	49	37
Stage 4	4	3
Average Age	57.9	58.6
PSA < 10 ng/mL	262*	139
PSA 10–20 ng/mL	21	15
PSA > 10 ng/mL	4	3
Annotation G3	N/A	293
Annotation G4	N/A	128
Annotation G5	N/A	17
Annotation Normal Gland	N/A	653
Annotation Tumor Stroma	N/A	514
Annotation Normal Stroma	N/A	168

Table shows the patient characteristic profile for the ELISA study and for the subset of patients in the TMA analysis. PSA: prostate specific antigen levels in serum at the time of prostatectomy. The * notes that one patient data point was not available for the PSA ng/mL value in the ELISA column. Every TMA annotation is the number of annotation types (G3, G4, G5, normal gland, tumor related stroma and normal related stroma) ascribed by the pathologist to regions of interest among the three stains visualized in serial section (perlecan, MMP-7, and β2M). Multiple annotations can exist in one tissue core. N/A: not applicable.

In analyzing these ratiometric values with regard to stage, perlecan stain concentration increased in tumor-related stroma with certain stages, as seen in Figure 2. A boxplot with log transformed values for TS/NS in comparison to the corresponding grades demonstrated that stage pT3a (invaded outside of the prostate capsule) had a statistically higher ratio of perlecan stain than pT2c and pT2a, even with low sampling size. Stage pT3a, however, was not significantly different from stage pT3b (invasion into seminal vesicles).

### Perlecan deposition is increased in G4 tissue

Perlecan stain in tissues was quantified for concentration as described in Materials and Methods, and normalized for each assigned Gleason grade in addition to the normal gland. Figure 3 shows the results comparing overall perlecan stain for all tissues assigned a grade of G4 versus the corresponding NG. In contrast to Figure 1, these results were analyzed over the concentration values for the entire pool of G4 tumors, and not normalized to cancer vs. normal tissue for each subject specimen. The quantification of perlecan stain in normal vs. G4 tissues is displayed in Figure 3A. Each dot represents a single specimen with the black bar as the mean and the gray bars as the standard deviation (SD) of the mean. The means differ with high significance (*p* value 0.0006) wherein G4 staining is higher for perlecan (20.15) than the normal gland (16.98). Figure 3B and 3C show representative images of perlecan stain (brown) with nuclear counterstain (blue) of normal (B) and G4 (C) prostate tissue. While the staining of the individual cells was low or absent, as expected for a secreted ECM protein, the immediately adjacent stroma around the cancerous lesions and the territorial matrix in between cells often showed increased perlecan staining (Figure 3C) over normal glands (Figure 3B). Staining showed a pericellular pattern of perlecan within the gland (arrow in 3C) that was largely absent in the normal glands (arrowhead in 3B). This result corroborates the data shown in Figure 2 where 71% of the G4 subjects had higher perlecan stain in cancer over their normal gland and a 44% increase in the mean ratiometric value. This demonstrates a trend toward increased perlecan expression in the stroma immediately surrounding the invading tumor.

### Perlecan and MMP-7 stain concentration are highly correlated in prostate tissue

**Figure 4:**
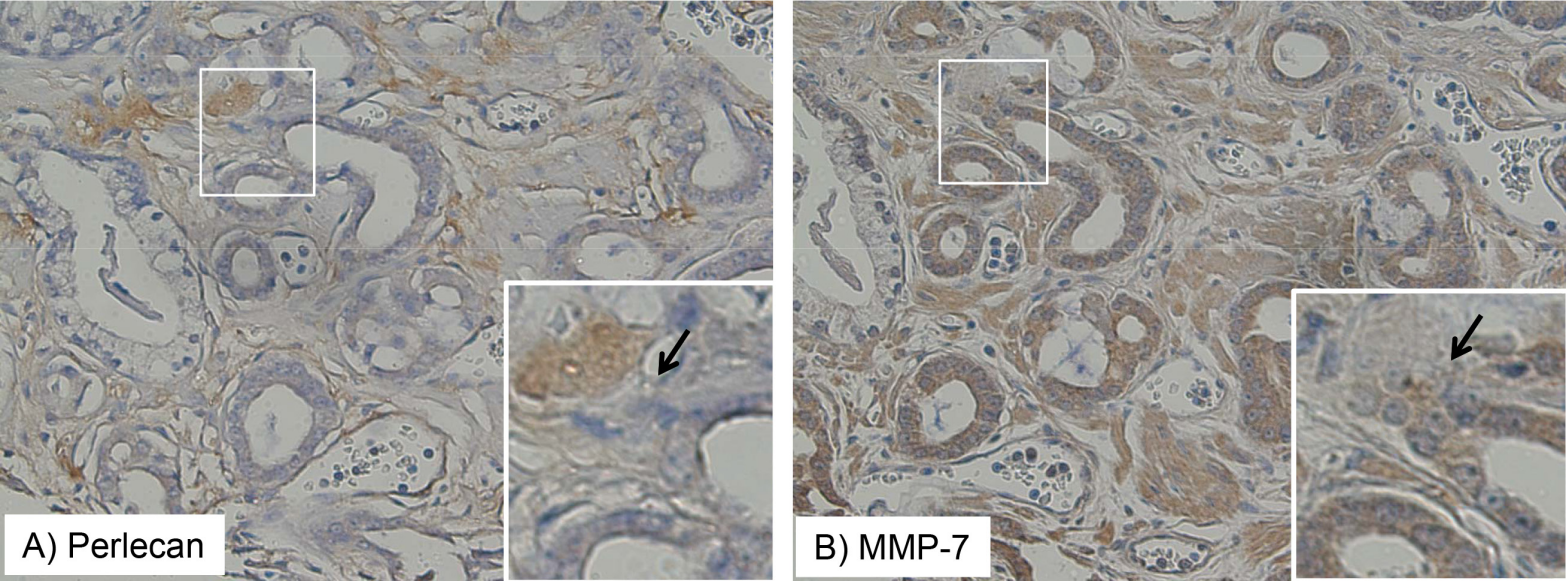
Perlecan and MMP-7 stain expression intersect in tissue microarray Staining patterns of perlecan (**A**) and MMP-7 (**B**) are shown in the same TMA tissue specimen in Gleason grade 3 tissue. The perlecan or MMP-7 antibody-detected stain is brown and the hematoxylin nuclear counterstain is blue. Perlecan and MMP-7 positively staining regions are adjacent to one another, but not overlapping, as indicated by the arrows.

We next wanted to determine if perlecan stain is correlated with MMP-7 stain, given that we know that MMP-7 efficiently proteolyzes the perlecan core protein [12]. Each cancer and normal tissue type was quantified for perlecan and MMP-7 stain concentration, normalized, and the correlation coefficients analyzed. Therefore, this correlation coefficient is not a direct measure of co-localization in series, but rather an index of whether within a given area of analysis of a tissue core, the levels of the two proteins are likely to be coordinately regulated. Table 2 provides Pearson's correlation coefficient values and associated significance (*p* values) for perlecan and MMP-7 staining in tissue sections with the annotations G3, G4, NG, and TS, and NS. In every annotation (save for G5, which was not analyzed due to low sample size) there was a positive correlation between perlecan and MMP-7 staining levels in the specimens. These correlation coefficients ranged from 0.45 (G3) to 0.65 (NS) with all significant *p* values less than 0.05. Therefore, perlecan and MMP-7 are statistically likely to be found together high or together low in the same encircled area of tissue, be it normal or cancerous, but do not typically directly overlap, which is to be expected if one is degraded by the other. Figure 4 shows a representative stain showing the directly appositional localization of perlecan (A) and MMP-7 (B) in one illustrative serially sectioned tissue specimen from the TMA in G3 tissue. The immunostained areas are shown in brown and the nuclei are stained blue. The arrows placed in approximately the same location in serial sections shown in Figure 4 indicate the junction where perlecan and MMP-7 intersect in the G3 tissue. While the regions of intense staining for the proteins do not overlap, they are found in the same area of the tissue, which can explain why the annotated stains are positively correlated with one another (positive correlation coefficients in Table 2).

**Table 2:** Correlation between overall perlecan and MMP-7 expression in specimens among various annotation types

Annotation Type	MMP-7 and Perlecan Correlation Coefficient	*p*-Value
Gleason Grade 3	0.45	**
Gleason Grade 4	0.46	*
Normal Gland	0.54	***
Tumor Stroma	0.48	*
Normal Stroma	0.65	***

The rho values (Pearson correlation coefficients) are given and analysis methods described in the text.

* Significance *p*-value codes: < 0.05

** < 0.01

*** < 0.001.

### Perlecan fragment and β2M variation in PCa subject serum

**Figure 5:**
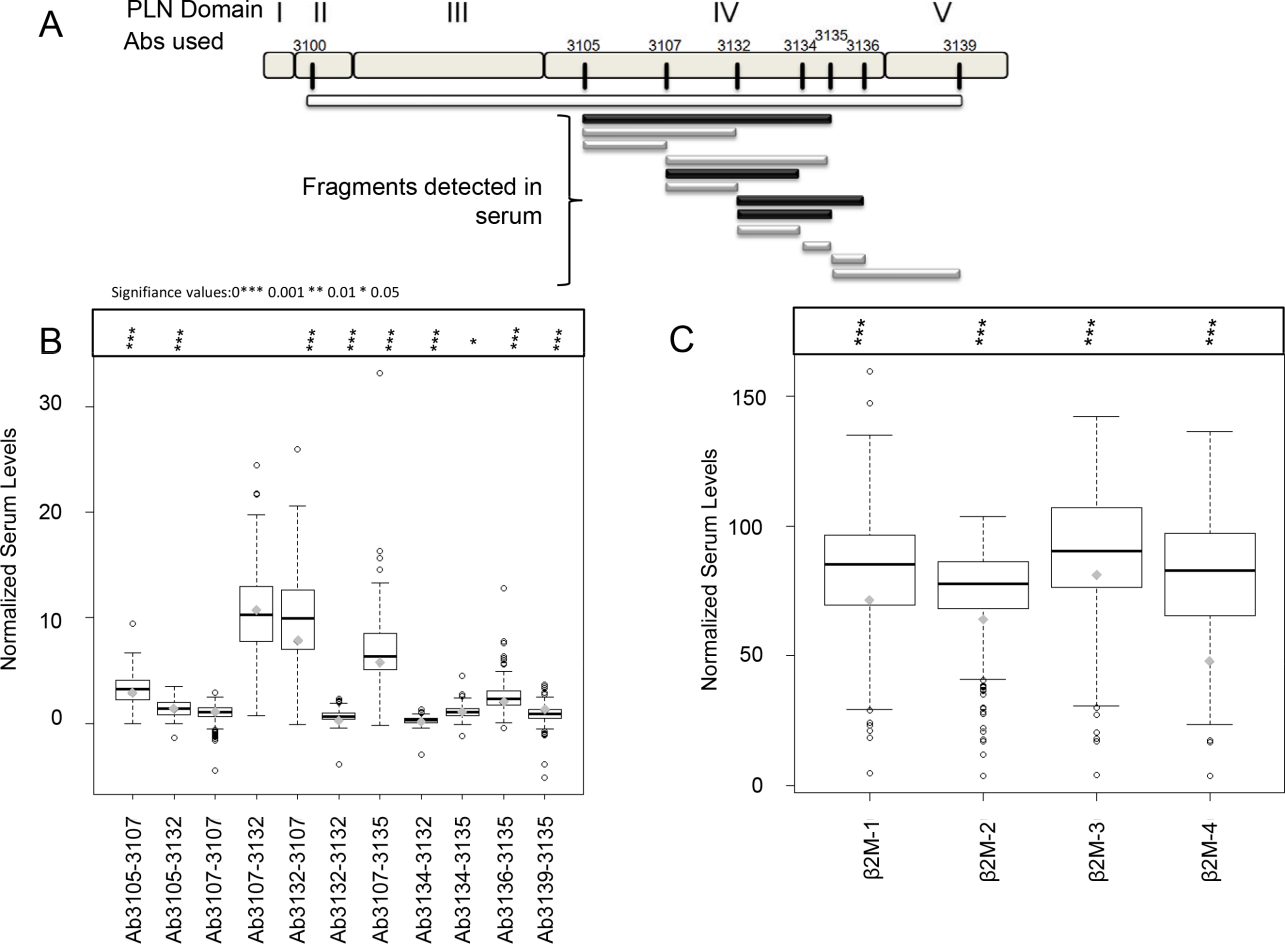
Sandwich ELISA detection of perlecan and β2-microglobulin (β2M) in serum (**A**) Schematic of perlecan core protein divided into domains I to V with the sites recognized by the eight antibodies used in the sandwich ELISA. The black and gray bars below show the detectable fragments (capture-detector pairs) from perlecan in patient sera. The white bar indicates full length perlecan was detectable by this assay in conditioned media of cells producing it, but not in patient sera. The fragments noted with gray bars were present in statistically significant levels from a pooled normal serum sample. (**B**) Box plot of observable capture-detector pairs in patient serum in comparison to a pooled normal serum sample (gray diamond) with associated *p*-values shown above. (**C**) Box plot with β2M serum values shown in comparison to a pooled normal serum sample (gray diamond) with associated *p*-values shown above. Student's unpaired *t*-test *p*-values: * < 0.05, *** < 0.001. Serum values were normalized to buffer control and negative control (non-immune rabbit immunoglobulin; see text).

Given the intersecting co-localization and statistical correlation of perlecan and its degrading protease MMP-7 in tissues, we next sought to determine if perlecan fragments potentially created by MMP-7 lysis of perlecan substrate in tissues could be detected in the serum of the same PCa patients and in serum of additional patients for whom we had serum collected at time of prostatectomy. Initial experiments (not shown) demonstrated that perlecan fragments can be measured in serum, do not bind to the fibrin clot, and do not need to be analyzed in plasma, which would be problematic for this analysis. To test for serum fragments of perlecan, we developed a capture-detector enzyme linked immunosorbent assay (ELISA) utilizing a series of paired peptide sequence-specific rabbit polyclonal antibodies that we developed across human perlecan's core protein spanning over 4,000 amino acids from domain II to domain V (Figure 5A). Intact perlecan and its fragments could be visualized by this method, spanning from peptide sequences recognized by antibodies 3100 to 3139 (“whole molecule”), in conditioned media of cells secreting full length unproteolyzed perlecan (Figure 5A, white bar). The far N-terminal region was omitted from this assay, as it is the site of attachment of the large GAG chains that sterically impede antibody access to the protein core. Every capture-detector pair that we employed produced signal when used to detect perlecan in conditioned media from cell lines producing large amounts of perlecan (positive control). When analyzing subject sera from prostatectomy patients, several antibody capture-detector pairs showed positive signal after normalizing for background buffer control only and non-immune rabbit IgG (Figure 5A, 5B). The fragments in serum that we detected are shown below the molecule in black and gray shaded lines spanning from the various perlecan-specific antibody capture-detector pairs. Assaying mouse serum did not produce a detectable signal (data not shown). Despite assaying over 288 patients (12 subjects were omitted from the final analysis for erroneous signal), positive signal from the N-terminal recognizing capture antibody 3100 was never found. Therefore, the N-terminal portion of perlecan could not be recognized, which suggests epitope destruction likely via proteolytic fragmentation or retention of this portion of the molecule in tissue. The most robust and common signal was derived from antibodies recognizing sequences in domain IV in perlecan. Only the most C-terminal domain V–IV antibody capture-detector pair, 3139–3135, in this assay produced a positive signal, pointing to enhanced cleavage at the C-terminus as well. Antibody pair 3136–3139 (the most C-terminal pair possible) was not included in the 288 subject assay, but it gave signal in preliminary experiments using a full 8 × 8 capture-detector array (data not shown). We were unable to perform an 8 × 8 capture-detector array for all samples because of the subject serum volume constraints. In comparison to a pooled normal serum control, some peptides detected with the shown capture-detector pairs were significantly elevated (*p* value < 0.05 and < 0.001) in cancerous sera over normal sera (gray diamond) using a student's *T*-test (Figure 5B). The domain V fragment (3139–3135), however, was slightly higher in pooled normal when compared to cancer samples. In Figure 5A the gray bars represent a statistical difference was found between the PCa subjects and the normal pooled serum. The black bars were present, but not significantly different from the pooled serum.

In addition to perlecan, β2M, a potential MMP regulator studied by our group, was analyzed in the same serum samples (Figure 5C) where β2M (1–4) was processed in conjunction with each of the four different perlecan detector antibodies (Biotinylated 3105, 3107, 3132, 3135). Thus, β2M levels were analyzed in quadruplicate per serum sample. Serum levels of β2M were found to be statistically higher than in a pooled normal sample (gray diamond) in each of the repeated assays (*p* value < 0.001).

### Levels of serum perlecan are associated with MMP-7 stain concentration

We next explored if any association existed between the MMP-7 expression in the tissue and its matched serum sample in terms of soluble perlecan fragments. The hypothesis being tested was that higher MMP-7 staining concentration would translate into greater proteolysis of any nearby perlecan at the “intersections” seen in the tissue arrays, and therefore would modulate the levels of circulating perlecan fragments. Overall MMP-7 staining concentration per patient was compared through analysis of co-variance (ANCOVA) with the perlecan capture-detector pairs. Table 3 displays the statistics derived from the ANCOVA analysis. Shown are the capture-detector pairs and related *F*-values and *p*-values. Four of the peptides identified using indicated capture-detector pairs shown in bold were found to be significantly associated with MMP-7 stain concentration. All of those perlecan peptides detected were derived from domain IV–IV antibody pairs, 3105–3102, 3107–3107, 3132–31312, and 3134–3132 with *p*-values less than 0.006. Therefore, overall MMP-7 prostate expression in cancerous subjects has an associative relationship with some portion of proteolyzed perlecan and associated fragment(s) present in serum.

**Table 3:** ELISA analysis to determine association of MMP-7 tissue staining concentration and perlecan fragments in serum

Perlecan Capture-Detector Pair	*F*-value	*p*-Value
Ab3105–3107	0.205	0.615
**Ab3105–3102**	**15.37**	**9.94E–05**
**Ab3107–3107**	**18.04**	**2.52E-05**
Ab3107–3132	2.745	0.0981
Ab3132–3107	1.328	0.25
**Ab3132–3132**	**8.135**	**0.0045**
Ab3132–3135	0	0.997
**Ab3134–3132**	**7.784**	**0.00545**
Ab3134–3135	0.141	0.708
Ab3136–3135	0.003	0.958
Ab3139–3135	1.921	0.166

The general model of association (analysis of co-variance, ANCOVA) between MMP-7 concentration stain and perlecan fragments in the ELISA assay was determined as described in the text. Reported are the *F* and associated *p*-values. Values in bold are considered significant (*p*-value < 0.05).

### Evidence of perlecan domain IV fragments in serum of stage IV PCa subjects

**Figure 6:**
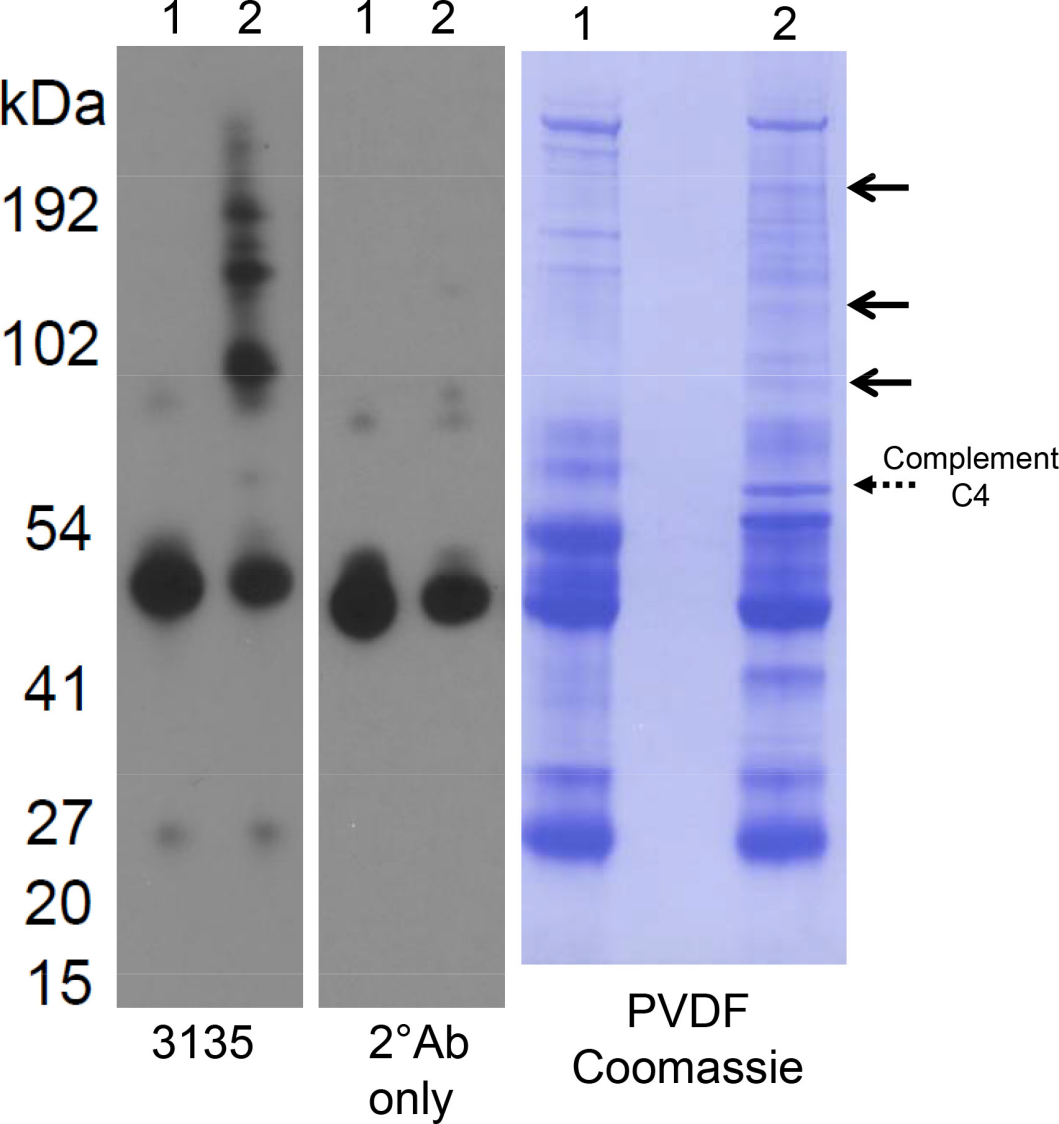
Western blot and protein stain of normal and stage IV prostate cancer sera Lane 1 contains four pooled normal sera and lane 2 contains four pooled stage IV prostate cancer sera. Both were non-specifically precipitated with protein AG agarose beads and processed by western blot with either the 3135 perlecan domain IV specific antibody (left) or secondary antibody alone (middle). On the left, the sera were transferred to a membrane blot and stained with Coomassie. The three top solid arrows represent the bands recognized by the 3135 anti-perlecan antibody. The bottom dashed arrow was not recognized by the 3135 antibody, but was present in cancer sera but not normal sera. N-terminal sequencing identified the dashed arrow band as complement factor C4 (see text).

This study largely focused on the tissue to serum relationship with regards to MMP-7 and its substrate, perlecan, at the time of radical prostatectomy and androgen withdrawal. None of the patients had known metastatic diseases at the time of resection. To examine if the fragments we identified were present in patients with metastatic disease, we conducted a small scale (4 subjects), study in which perlecan levels were assayed by western blot using the 3135 antibody in a pooled stage IV PCa serum samples and a normal age/sex matched pooled control as seen in Figure 6. Lane 1 shows the results of analysis of normal sera and lane 2 shows the stage IV PCa sera. Attempts were made to immunoprecipitate with the perlecan specific antibody 3135, but the same results were obtained regardless of whether the protein AG magnetic beads only, non-immune rabbit IgG or the 3135 antibody were used as the precipitation agent (not shown). While not useful in immunoprecipitation, the 3135 antibody worked very well in detecting differences in perlecan fragments between the normal and stage IV sera in western blots. Three primary bands (100 to 200 kDa range) were found in the stage IV sera that are not present in the normal sera. The goat anti-rabbit secondary antibody by itself failed to detect these bands. The secondary antibody only detected the IgG heavy chain protein at 50 kDa. Transfer to a membrane and Coomassie staining highlighted the differences in the bands between normal and cancer sera that were positive for the 3135 antibody reactivity (right arrows). Attempts to identify the bands by N-terminal sequencing were unsuccessful because of the high molecular weight resulting in low picomolar amounts of protein that were present. The dashed arrow above 54 kDa that was not recognized by the perlecan antibody was identified as complement factor C4. Nevertheless, these data demonstrate that as the disease progresses toward metastasis, new perlecan fragments in serum appear.

## DISCUSSION

Dynamic changes in the cancer and the stroma surrounding PCa foci play major roles in determining the course of the disease, including its ability to invade local connective tissue. Perlecan is a key component of the reactive stroma [5] that must be removed for cancer cells to invade and colonize nearby tissue. This study sought to reveal the relationship of levels of perlecan, perlecan fragments, the perlecan degrading metalloproteinase MMP-7, and a multifunctional tissue factor and activator of metalloproteinase, β2M, in PCa tissue and serum collected at the time of prostatectomy from a large 288 patient cohort. Retrieval conditions were improved to allow visualization of extracellular deposited perlecan in paraffin-embedded blocks, unlike a previous study that only revealed largely intracellular signal in PCa samples [19]. This allowed us for the first time to systematically analyze cancer-associated, cancer-associated and normal stroma, and extracellularly deposited perlecan in organ confined PCa as a potential index of grade and likelihood of tissue invasion.

A clear observation from this work is that the levels of perlecan were higher, i.e. more concentrated, in many G3 and especially G4 prostatectomy tissues and surrounding stroma, when compared to regions of normal gland. Perlecan is a highly conserved growth factor-binding component of ECM that also plays a key role in separating cell layers in normal tissues [4]. Earlier knockdown studies showed that loss of perlecan decreased the overall tumor growth and tumor vascularization [25]. The increased levels of perlecan seen in G4 tissues and stroma thus may indicate that increases in perlecan expression in the tumor microenvironment are part a tissue reaction to the presence of the cancer, and that increases in expression in the cancer cells may be part of an adaptive survival and growth response. Extending our previous observations [5], increased perlecan expression in the desmoplastic stroma within individual PCa patient cores was associated with higher stage, indicative of higher tissue invasive capability.

We recently showed that MMP-7 efficiently degrades perlecan in its native context [12]. In this study, we found that perlecan stain was positively correlated with MMP-7 stain and both typically were found in apposition to one another. This relationship has been reported in dysplasia of oral mucosa and oral carcinoma *in situ*, wherein perlecan and MMP-7 were commonly juxtaposed [26]. Therefore, perlecan upregulation and subsequent MMP-7 mediated destruction may be a common feature among locally invasive cancers. This idea is consistent with our findings that MMP-7 and perlecan immunostaining levels, together high or together low, were highly correlated with each other in individual tissue specimen cores. The co-expression of perlecan and MMP-7 that we observed in normal tissue (0.67 Pearson coefficient) can be explained if endogenous inhibitors (TIMP-1) are expressed along with MMP-7 as has been reported [17]. In cancerous tissue, MMP-7 expressing cells can degrade perlecan in the matrix, and, interestingly, it has been shown that certain HS moieties present on perlecan can bind and activate MMP-7 [27], creating a positive “feed forward” response that could speed local tissue invasion. Other proteoglycans (e.g. syndecan-1 and glypican-1) show positive correlation with PCa and also may be proteolyzed by activated MMP-7 [28]. It is worth considering that some patients who are characterized as “superexpressers” of MMP-7 (high ratiometric levels of MMP-7, see Figure 2) in regions of high perlecan expression could be considered of higher grade than those without this condition, and that such patients might be at higher risk of rapid disease progression. Unfortunately, the number of patients having a biochemical relapse during this prostatectomy study was insufficient to determine if this is the case.

Perlecan and β2M co-localize in hemodialysis induced β-amyloidosis where perlecan acts as an early driver of fibrillogenesis [29]. Additionally, β2M increases the production of MMPs but not the corollary TIMP in human synovial fibroblasts [22]. We considered that perhaps a positive association could exist for MMP-7 and β2M in prostatectomy specimens. However, co-localization was not observed in this specimen set. While perlecan and MMP-7 levels were positively correlated in tissue, β2M levels were not correlated directly with either in tissue specimens. Thus, it is unlikely that β2M is a direct modifier of perlecan-MMP-7 driven tissue invasion. β2M was observed by serum ELISA to be increased in patients with PCa. This increase is consistent with our earlier work defining β2M as a soluble growth factor driving osteomimicry, PCa progression and growth [23]. Findings here, however, indicate that, at least within this 288 large patient cohort that we examined, while β2M is increased in serum of prostatectomy patients, it does not have a significant correlative relationship with MMP-7 or perlecan either in tissue or serum, a result consistent with the presence of β2M in the inflammatory processes, and its participation in multiple functions related to cancer and normal physiologic response to inflammation.

Reasoning that the destruction of matrix perlecan by MMP-7 at borders should be detectable by the presence of perlecan-derived fragments in serum, we examined serum from the full 288 prostatectomy patient cohort. By creating a panel of antibodies recognizing perlecan from one end to the other and using a reliable automated capture-detection epitope mapping assay, we detected fragments in patient sera largely derived from the 21 Ig repeat domain IV of this protein that spans almost half of the molecule [30]. This was not entirely unexpected given the domain's length and the existence of multiple predicted and demonstrated sites for proteolysis in this region [31]. The epitope mapping assay would have identified full length perlecan if it were present in serum, as positive signal for nearly the whole molecule (domain II to domain V) was found in conditioned media of perlecan secreting cells and when purified full length perlecan was added to the assays. Thus, the inability to detect full length perlecan in the sera of prostatectomy patients indicates that tissue perlecan must be at least partially degraded to enter the serum. Also, the pattern of perlecan peptide in serum that we detected indicates that perlecan cleavage occurs predominately from the C-terminus, which is consistent with our previous finding that MMP-7 cleaves domain IV inward from the C-terminus *in vitro* [12].

Interestingly, levels of some perlecan capture-detector pairs or fragments were significantly increased in prostatectomy patients over those in pooled normal sera, which is consistent with increased perlecan turnover during cancer invasion of tissue. This is true despite the fact that perlecan destruction and release into serum can occur during atherosclerotic associated intimal hyperplasia [32], arthritis [33], or any other inflammatory or acute wounding condition that destroys perlecan-rich joint tissue [34]. Nonetheless, the finding that specific perlecan fragments were elevated in serum in conjunction with MMP-7 elevation in tissue suggests that there are fragments derived from perlecan that may be useful indices of cancer tissue invasion. Current studies in our laboratories seek to develop useful and specific probes for MMP-7 elicited degradation fragments in tissue.

Because of its association with tissue destruction, we suspected patients with active bone metastases also would have elevated perlecan fragments in serum. None of the 288 patients in the original prostatectomy cohort had detectable metastasis at the time of surgery, so this question was addressed with a separate, smaller, serum pool from patients with active metastatic disease. The domain IV antibody, 3135, was used for this study because it consistently recognizes perlecan in a variety of preparations [12]. Western blot analysis revealed that several large perlecan antibody reactive fragments (100–200 kDa in size) were present in patient serum with active metastasis, but not in normal serum. Full length perlecan core protein (467 kDa without GAG chains) typically would be near the top of the gel in this system. One band visible by Coomassie stain also was clearly present in sera from PCa but not normal sera, although this band did not react with antibody 3135. Mass spec analysis identified this band as complement C4, a protein reported decades ago to be upregulated in prostatic fluid of PCa patients [35]. Future studies are needed to examine the fragment contribution from the primary PCa versus the active metastasis turnover in the bone or if different perlecan fragments track to different tumor sites. The resolution of our ELISA assay we used is insufficient to identify subtly different fragments from the same region of perlecan, as seen in Figure 6. Nevertheless, the presence of perlecan-derived fragments in PCa patient serum indicates that it might be a useful index by which to suspect that PCa patients have relapsed or have early metastasis. Perlecan is highly expressed in the bone marrow reticular matrix and reports show that higher MMP-7 serum concentrations are associated with metastatic disease and are predictive of poor prognosis [36]. Future studies could determine if the slope or velocity of perlecan fragment changes in serum that are created as a consequence of MMP-7 activity couples with the current prostate specific antigen (PSA) velocity in detecting early metastatic lesions.

In summary, examination of a large cohort of 288 prostatectomy patient samples showed that the presence of MMP-7 in tissue blocks assessed by IHC correlated with the presence of specific perlecan fragments in serum. Overall, we found that the enzyme-substrate relationship between perlecan and MMP-7 predicted *in silico*, confirmed *in vitro* [12, 31], and now shown to extend to both tissue and serological patient specimens (this study), may be a key component of PCa tissue invasion. Perlecan concentration in the entire TMA was upregulated in PCa G3 and, especially, G4 and MMP-7 and perlecan were highly correlated in prostatectomy tissue specimens. Additionally, an association exists between MMP-7 staining level and the presence of perlecan fragments even in organ confined disease. While this study focused on MMP-7 staining amount in relation to perlecan, other MMPs and even other classes of cancer-associated proteases (i.e. membrane type 1 and 2 MMP) almost certainly contribute to the proteolysis of perlecan during cancer invasion of tissue [14, 37]. Taken together, this study indicates that MMP-7-associated degradation of perlecan-rich matrices surrounding PCa cells occurs during tissue invasion, creating fragments that can enter the circulation along with other local modulators of tissue homeostasis including β2M. The utility of using these perlecan fragments and β2M as independent PCa biomarkers for invasive or recurrent disease merits further investigation.

## MATERIALS AND METHODS

### Tissue and serum procurement and processing

All tissue and serum specimens were collected with consent under institutional IRB approved protocols at the Winship Cancer Center, Emory University, Atlanta, GA. Samples were collected at the time of radical prostatectomy after a PCa diagnosis. Serum samples were centrifuged at 2,400 × g for 10 min at 18°C. The serum supernatant was removed, and stored in aliquots at −80°C until used. Tissue samples were fixed in formalin and embedded in paraffin. From each tissue block a region of cancerous and normal tissue core of 0.6 mm were removed and inserted into a recipient block. Tissue cores were obtained using a thin-walled, sharpened stainless steel tube instrument (Beecher Instruments, Silver Spring, MD USA). Recipient array paraffin blocks were sectioned at 5 μm onto glass slides for subsequent IHC.

### Subject population

Table 1 describes the population characteristics of PCa subjects participating in this study. The test population consisted entirely of men undergoing prostatectomy in whom cancers usually are more indolent. None of the subjects had observable or known bone or other soft tissue metastases at the time of surgery. Nevertheless, a subset of subjects experienced biochemical relapse/recurrence during the follow up period. Sera from 288 patients were analyzed and approximately half of these subjects (157) were used for the TMA. It thus was possible to match the serum data to the IHC data for each subject in this study. Normal serum was pooled from 12 healthy male adult volunteer subjects and provided as a custom product by SeraCare (Milford, MA). Supplementary Table S1 provides the overall accounting of pathologist encircled annotations/tissue types and their associated stage. Multiple annotations can exist in the same subject and the same accounting was used for three stains as described below.

### Development of custom antibodies

The rabbit anti-human perlecan antibodies were generated using a proprietary technology developed at Strategic Diagnostics, Inc. (Newark, DE). The eight genomic antibodies chosen for this study were designated 3100, 3105, 3107, 3132, 3134, 3135, 3136, and 3139 (see Figure 5 for locations on perlecan core protein). All are rabbit polyclonal antibodies that recognize unique and regularly spaced 100 amino acid perlecan sequences spanning the length of the molecule. Additionally, the rabbit polyclonal β2M antibody used in the ELISA was generated using the same proprietary technology. All antibodies are the property of Strategic Diagnostics, Newark DE.

### Other antibodies

The antibodies used for the TMA were the rabbit anti-HSPG2 prestige antibody (Sigma-Aldrich, St. Louis, MO), the mouse anti-MMP-7 ID2 antibody (EMD Millipore, Billerica, MA), and the rabbit anti-β2M antibody (Dako, Glostrup, Denmark). The polyclonal rabbit anti-GFP antibody (cat. No. 2555S) was purchased from Cell Signaling Technologies (Beverly, MA).

### TMA staining and quantification

Staining for perlecan was performed as described previously [5]. MMP-7 and β2M also were stained with variation in antigen retrieval method as noted below. Briefly, tissues were deparaffinized with xylene and graded ethanol, incubated with hydrogen peroxide in methanol, washed, and subjected to antigen retrieval. Perlecan was retrieved with bovine testicular hyaluronidase (Sigma-Aldrich) for 30 min at room temperature (RT), MMP-7 was retrieved with Diva Decloaker (Biocare Medical, Concord, CA) for 30 min in a steamer, and β2M was not retrieved per manufacturer instructions. Following blocking in Background Punisher (Biocare), tissue was incubated with primary antibody (anti-HSPG2, 1:100; anti-MMP-7, 1:200; anti-β2M, 1:800) overnight at 4°C. The staining was visualized with Diagnostic Biosystems' (Pleasanton, CA) Polymer Penetration Enhancer, anti-mouse/rabbit PolyVue horseradish peroxidase, and (3, 3′-diaminobenzidine) DAB chromogen. Nuclei were counterstained with CAT hematoxylin (Diagnostic Biosystems) and a sodium bicarbonate bluing agent. Tissue was dehydrated with graded ethanol and xylene and mounted in Cytoseal 60 (Fisher Scientific, Hampton, NH).

Each stained slide was imaged and quantified with a Leica Biosystems (Buffalo Grove, IL) instrument and software. For each core, a certified pathologist identified and encircled several annotation types if present: NG, G3/4/5, NS and TS. These regions of interest were quantified for “light intensity transmission” (the lower the light transmission the higher/darker the actual stain amount) by the Leica Biosystem software and converted to “concentration values” with a formula including absorbance and pixel area. Instead of using both values in this study we determined that light transmission values and concentration values were correlated statistically to be inverse with a Pearson coefficient of −0.89 and a *p* value of 2.2E–16 (Supplementary Figure 3). Therefore, we could reliably use one value for this study. Concentration values were used in the detailed statistical study (see below) because concentration values actually increases with the amount of stain (darker) which makes understanding the associations with other factors such as serum protein quantities more straightforward.

### Sandwich ELISA

All eight custom anti-perlecan antibodies, β2M antibody, and non-immune rabbit IgG negative control antibodies were printed in triplicate onto Whatman 16 Pad nitrocellulose Fast Slides (GE Healthcare, Pittsburgh, PA) with an Arrayit NanoPrint^®^ (Arrayit, Sunnyvale, CA) instrument according to manufacturer's specifications. Slides were placed in Whatman Fast Slide Frames (GE Healthcare) capable of isolating each nitrocellulose pad for individual processing. All steps were performed at RT on a 120 rotations per min (RPM) shaker unless otherwise stated. Pads with printed antibodies were blocked with Blockit^®^ Blocking Buffer (Arrayit cat no. BKT) for 1 hr and washed 6 times with PBST. PCa subject serum and normal serum was diluted by a third in PBST (phosphate buffered saline, 0.05% (v/v) Tween-20) (Sigma-Aldrich). PCa cell line PC-3 (American Type Culture Collection (ATCC), Manassas, VA, cat no. CRL-1435) in serum free conditioned media (F-12K (ATCC) medium with 1X penicillin/streptomycin (P/S) (Life Technologies, Carlsbad, CA) was diluted by half in PBST for a positive control consisting of full length perlecan. The samples were placed into eight separate pads, incubated for 1 hr and then washed 6 times with PBST. Detector antibodies conjugated to biotin were incubated individually on separate pads at 2 μg/mL in PBST for 1 hr followed by 6 PBST washes. Pads were incubated with 1 μg/mL streptavidin Cy-5 (GE Healthcare) in PBST for 1 hr. Following 6 PBST washes, pads were removed from cassettes, spin dried and scanned at 633 nm with a LS Reloaded^™^ Series Microarray Laser Scanner (Tecan, Mannedorf, Switzerland) and analyzed with Array Pro^®^ Analyzer software. Intensity values were normalized to account for general background by subtraction. Background subtracted values were divided by buffer control (same pad, same spot) to control for pad to pad variability. This value then had the negative control rabbit IgG values subtracted from it. Therefore, the assay may produce negative values for analysis.

### Western blot and coomassie stain of subject serum

Serum samples were obtained from Bioreclamation, Inc. (Baltimore, MD). Four age and sex-matched normal sera (HMSRM BRH506649-52) and four stage IV PCa sera (HMSRM-PROSTATE BRH506645-48) were analyzed. Aliquots of each subject were thawed, combined (320 μL total), Protease Inhibitor Cocktail added (Pierce, Rockford, IL) and diluted with PBS. Protein AG magnetic beads (25 μL) (Chromatrap^®^, Ashland, VA) were added to the mixture and allowed to rotate end over end for 1 hr at 4°C and 30 min at RT. Sera was removed and the beads washed 4 × with PBS. After washing, 20 uL of PBS were added along with 5 × SDS-reducing sample buffer (Pierce) and heated to 99°C for 10 min. The supernatant was removed and separated with 4–12% (w/v) polyacrylamide SDS-PAGE in MOPS buffer (Life Technologies) and transferred to nitrocellulose (BioRad, Hercules, CA) in Tris-glycine buffer at 4°C at 40 volts for 5 hrs. Membranes were blocked in 3% (w/v) BSA Tris buffered saline with 0.05% Tween-20 (TBST) for 2 hrs at RT. Antibody 3135 (1:10,000) was added to the block solution overnight on a 4°C shaker. Following three by 5 min TBST washes, the membranes were incubated with 1:200,000 goat anti-rabbit HRP conjugated antibody in 3% BSA TBST for 2 hr at RT. Blots were washed again and incubated with chemiluminescence substrate (West Dura extended substrate, Pierce) for 5 min and exposed to film. For the Coomassie stain and N-terminal sequencing the protein was transferred to a methanol pre-soaked 0.22 μm pore polyvinylidene fluoride (PVDF) membrane in 25 mM Tris, 192 mM glycine buffer with 20% (v/v) methanol at 50 V for 5 hrs at 4°C. The PVDF membrane was washed with distilled water several times to remove glycine background, and dipped in methanol again. The membrane was stained with 0.1% (w/v) Coomassie Blue R-250 in 40% methanol, 1% (v/v) acetic acid for 1 min, and destained with 50% methanol in water solution. After extensive MilliQ water washes, the membrane was left to air dry. The membrane was sent to Midwest Analytical Inc. Protein Sequencing (St. Louis, MO) for N-terminal Edman degradation protein sequencing.

### Statistical analysis

Each statistical model utilized is described specifically along with the results of each study. In general we used a linear mixed-effect model to compare differences between and among groups. Staining concentration values were normalized by taking the square root. For strength of correlation, the Pearson correlation coefficient was determined. Significance was set a priori at *p* < 0.05.

## SUPPLEMENTARY MATERIALS FIGURES AND TABLE


