# Correction: Perlecan/HSPG2 and matrilysin/MMP-7 as indices of tissue invasion: tissue localization and circulating perlecan fragments in a cohort of 288 radical prostatectomy patients

**DOI:** 10.18632/oncotarget.11976

**Published:** 2016-12-09

**Authors:** Brian Grindel, Quanlin Li, Rebecca Arnold, John Petros, Majd Zayzafoon, Mark Muldoon, James Stave, Leland W. K. Chung, Mary C. Farach-Carson

Present section: Due to an error made during submission of the revised files, acknowledgements were omitted from this manuscript.

Correct section: Corrected acknowledgements information is provided below. Authors sincerely apologize for this oversight.

Original article: Oncotarget. 2016; 7(9):10433-47. doi: 10.18632/oncotarget.7197.

