# Supplementary material for: Perlecan/HSPG2 and matrilysin/MMP-7 as indices of tissue invasion: tissue localization and circulating perlecan fragments in a cohort of 288 radical prostatectomy patients
Source: Oncotarget. 2016 Feb 4;7(9):10433–47. doi: 10.18632/oncotarget.7197 (PMC4891130; doi:10.18632/oncotarget.7197)
Supplement: Supplementary file 1 [file oncotarget-07-10433-s001.pdf]

Perlecan/HSPG2 and matrilysin/MMP-7 as indices of tissue invasion: tissue localization and circulating perlecan fragments in a cohort of 288 radical prostatectomy patients

Supplementary Materials

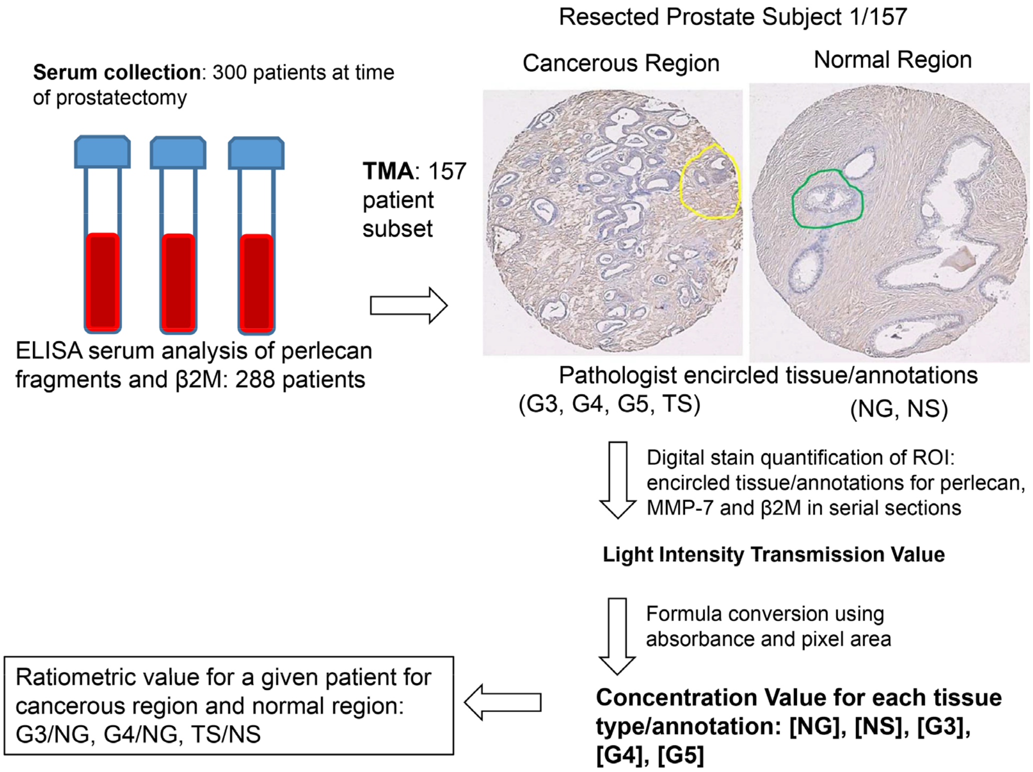

Supplementary Figure S1: Study design. In this study, serum was collected from 300 patients at the time of prostatectomy. These sera were analyzed for perlecan fragments and β2M levels (288 samples total, used in Figure 5 and Table 3). A subset of these patients (157) were used to construct a serialized tissue microarray (TMA). Each subject had a corresponding cancerous core and normal adjacent core. After digitization, a pathologist ascribed a region of interest (ROI) consisting of different annotation/tissue types (Gleason grade 3, 4, 5 (G3, 4, 5), normal gland (NG), normal stroma (NS), tumor-related stroma (TS)). These ROIs were quantified to provide a light intensity transmission value. Through a formula accounting for pixel area and absorbance, the value was transmuted into a concentration value (concentration values used in Figure 3, Table 2 and 3). In some analyses, cancer cores and normal cores within each patient were compared to derive new ratiometric values, G3/NG, G4/NG, and TS/NS (as seen in Figures 1 and 2). This provides a new statistic that depicts how much higher the given stain is within each subject in comparison to its normal counterpart.

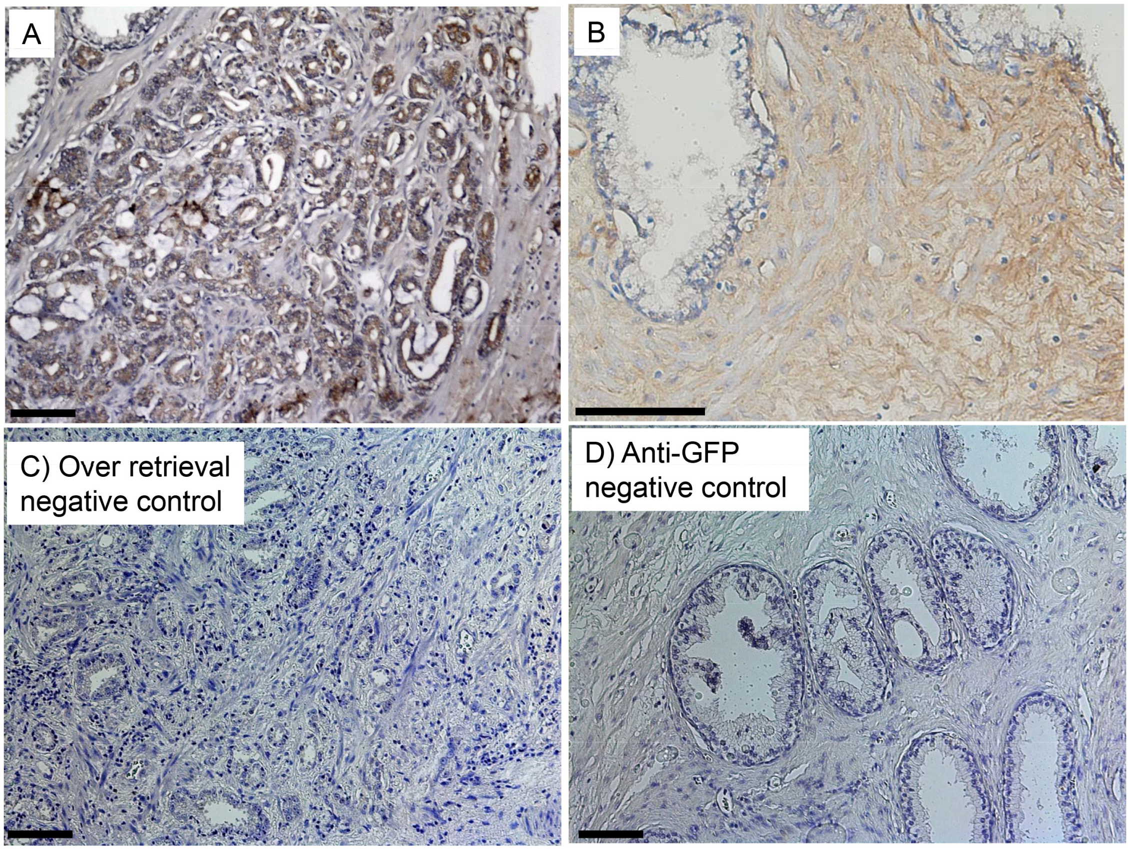

Supplementary Figure S2: Perlecan antibody validation. Perlecan was detected in tissues as described in the text (stained brown) and shown with a hematoxylin nuclear counterstain (blue) in tissues. (**A**) A positive stain for glandular perlecan in cancer. (**B**) Stromally deposited perlecan with absence in the glands. Other controls used in this study: (**C**) over digestion with proteinase K destroyed perlecan epitopes and produced a negative background control for IHC protocol; (**D**) negative/background stain with an anti-green fluorescent protein antibody. Scale bar is 100 μm.

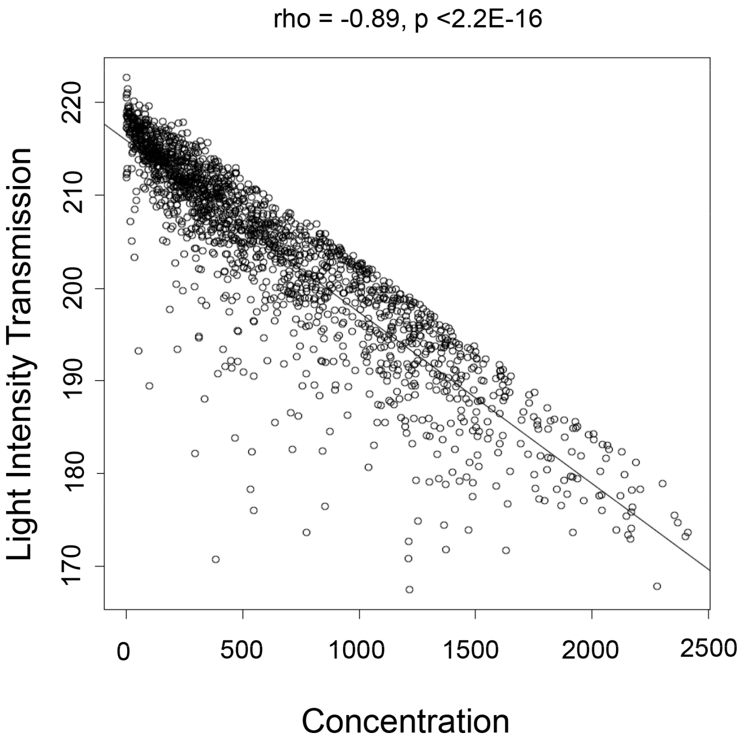

Supplementary Figure S3: Tissue staining light transmission and calculated concentration values correlate inversely. For each antibody stain (perlecan, MMP-7, and β2M), quantified staining light transmission values (y-axis) were plotted against the concentration values (x-axis) that are calculated from light transmission and other quantified values. "Light intensity transmission values" actually decrease with enhanced/darker staining and "concentration values" mostly increase with enhanced/darker staining. To determine if we needed to use both or just one of the values in this study we calculated their correlation. Utilizing Pearson correlation, an inverse relationship exists with a rho value of −0.89 and *p*-value of 0.22E–16. Since they are highly negatively correlated, only one value is needed. Concentration values, rather than light intensity transmission values, ultimately were used in the study because they increased with greater stain amount (see text).

Supplementary Table S1: Tissue microarray pathologist circled annotations/tissue types within each stage

Annotations	pT1a	pT2a	pT2b	pT2c	pT3a	pT3b	pT4	Total
**G3**	0	15	24	192	30	15	7	293
**G4**	0	7	4	45	39	31	2	128
**G5**	0	0	0	0	5	11	1	17
**NG**	3	41	83	375	104	41	6	653
**NS**	6	37	72	287	69	29	14	514
**TS**	0	9	10	83	30	35	1	168

Annotations/tissue types are across all three antibody stains
